# ﻿Updated checklist, habitat affinities, and changes over time of the Indiana (USA) caddisfly fauna (Insecta, Trichoptera)

**DOI:** 10.3897/zookeys.1216.129914

**Published:** 2024-10-25

**Authors:** David C. Houghton, R. Edward DeWalt

**Affiliations:** 1 Department of Biology, Hillsdale College, 33 East College Street, Hillsdale, MI 49242, USA Hillsdale College Hillsdale United States of America; 2 Illinois Natural History Survey, 1816 South Oak Street, Champaign, IL 61820, USA Illinois Natural History Survey Champaign United States of America

**Keywords:** Biological diversity, conservation, distribution, insect, Upper Midwest

## Abstract

Based on recent collecting and a synthesis of ~100 years of historical data, 219 caddisfly species are reported from the state of Indiana. Seventeen species are reported herein from the state for the first time, including two previously thought to be endemic to the southeastern USA. Species records are also presented herein organized by drainage basin, ecoregion, glacial history, and waterbody type for two distinct time periods: before 1983 and after 2005. More species were reported from the state before 1983 than after 2005, despite collecting almost 3× the number of occurrence records during the latter period. Species occurrence records were greater for most families and functional feeding groups (FFGs) for the post-2005 time period, although the Limnephilidae, Phryganeidae, Molannidae, and Lepidostomatidae, particularly those in the shredder FFG, instead had greater records before 1983. This loss of shredders probably reflected the ongoing habitat degradation within the state. While species rarefaction predicts only a few more species to be found in Indiana, many regions still remain under-sampled and 44 species have not been collected in >40 years.

## ﻿Introduction

The caddisflies (Trichoptera) constitute an important group of aquatic organisms due to their high overall abundance, high species richness, high ecological diversity, and differing sensitivities to various anthropogenic disturbances (Barbour et al. 1999; Morse et al. 2019). Determining caddisfly distributions and habitat affinities, therefore, is valuable for assessing water quality and other aspects of ecosystem integrity (Dohet 2002; Houghton and DeWalt 2021). Assessing changes in such data over time can be especially valuable (Houghton and Holzenthal 2010).

The caddisflies of the Upper Midwest region of the United States (MAFWA 2023) have been studied for nearly 100 years, starting with the Illinois fauna (Ross 1938, 1944), including more recent comprehensive studies of Kentucky (Floyd et al. 2012), Michigan (Houghton et al. 2018, Minnesota (Houghton 2012), Missouri (Moulton and Stewart 1996), Ohio (Armitage et al. 2011), and Wisconsin (Hilsenhoff 1995), and culminating with an overall checklist of the entire region (Houghton et al. 2022). The last paper included 131 new state species records combined from eight different states, including five from Indiana, demonstrating that even well-collected areas still contain undiscovered species.

Research on the Indiana caddisfly fauna encompasses two approximate time periods. The first period began in the 1930s and concluded with Waltz and McCafferty’s (1983) checklist of 190 species. Specimens from this period are housed primarily in the Purdue University Entomological Research Collection (PERC) and the Illinois Natural History Survey Insect Collection (INHS). After a ~20-year pause, caddisfly collecting renewed in the early 2000s with subsequent studies by DeWalt et al. (2016a) and Bolton et al. (2019), as well as many specimens accessioned into the PERC, INHS and, more recently, the Hillsdale College Insect Collection (HCIC). This nearly 100-year collecting history provided an opportunity to assess any changes in the caddisfly fauna over time.

**Figure 1.**
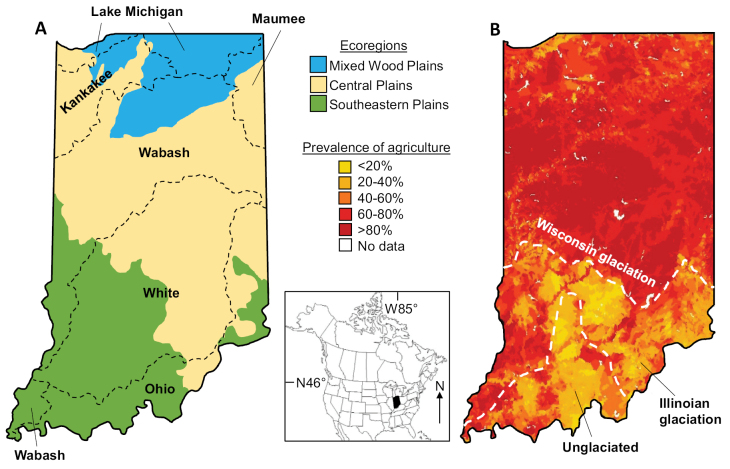
Location of the state of Indiana showing the approximate boundaries of drainage basins and ecoregions (**A**), and prevalence of agriculture and Pleistocene glacial history (**B**).

Indiana is composed of a single USEPA Level I ecoregion and three secondary ecoregions: Central Plains, Mixed Wood Plains, and Southeastern Plains (Fig. 1). The predominant land use is agriculture in the form of row crops and pasture, especially in the northern two thirds of the state. Land use corresponds strongly with glacial history, as the low-gradient environments and abundant glacial till of the more recent Wisconsin glaciation are more conducive to farming than the higher-gradient and more eroded older landscapes of the Illinoian glaciation and unglaciated regions.

The primary objective of this study was to update the state caddisfly checklist for Indiana and relate the occurrences of all species to drainage basin, ecoregion, glacial history, and waterbody type. We also assessed the rarity of all Indiana species. Since >40 years had passed since the last state checklist, we assessed any notable changes to the fauna during this period. Further, we used species rarefaction to predict total species richness for the state and assessed the importance of collecting effort on a regional level.

## ﻿Materials and methods

**Figure 2.**
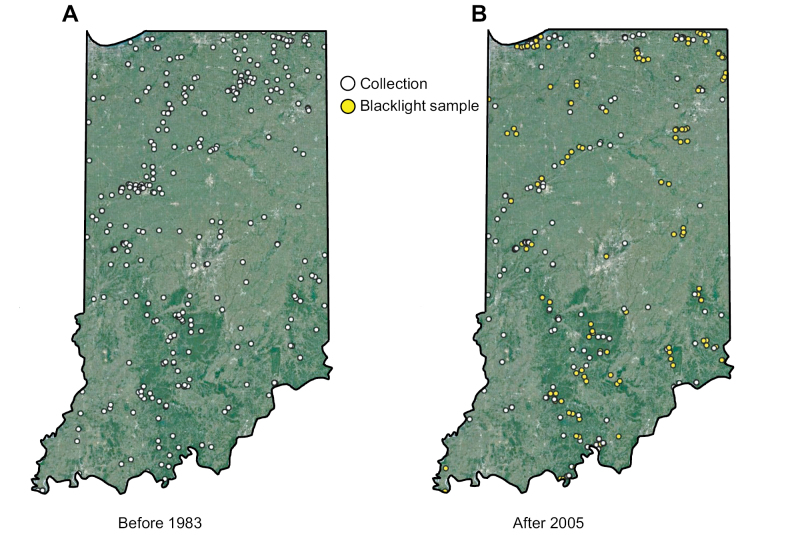
Collecting localities of Indiana caddisflies before 1983 (**A**) and after 2005 (**B**). White markers represent collections of unknown sampling effort whereas yellow markers represent ~2 h ultraviolet blacklight samples. Base map © Google, NOAA.

Our primary sampling devices included two types of ultraviolet light traps: an unattended 8-watt light placed over a white pan filled with ethanol, and an attended 12-watt light suspended from a white sheet with two pans filled with ethanol at its base. Such devices were set out at dusk near aquatic habitats and retrieved approximately two hours later (Houghton 2004; Wright et al. 2013; DeWalt et al. 2016a). The nocturnally active caddisfly adults were attracted to the lights and either fell into the pan or were hand-collected (Fabian et al. 2024). Sampling the winged adults is necessary for taxonomic and conservation studies since, unlike larvae, they are usually identifiable to the species level. Moreover, since adults are attracted to lights irrespective of their specific natal microhabitat or functional feeding group (FFG), inferences on ecology and biotic integrity can be made about an ecosystem without the sampling bias that affects benthic studies (Cao and Hawkins 2011). We and our colleagues collected 194 of these ultraviolet light samples from 2005–2023 (Fig. 2, Suppl. material 1). We also databased specimens from the INHS and PERC going back to the early 1900s. These specimens represented collections of unknown effort. Thus, Fig. 2 makes the distinction between “collections” (unknown effort) and “samples” (the ultraviolet light sampling regime described above). All specimens are housed in either the HCIC, INHS, or PERC institutional collection.

We associated all 1116 unique collecting localities with drainage basin, ecoregion, glacial maximum, and waterbody type. Our approach for dividing the state into geographic and ecological regions was a balance between having divisions specific enough to reflect biological differences, yet large enough to maintain a consistent collecting effort between them. Thus, we divided the state by United States Environmental Protection Agency Level II ecoregions (https://www.epa.gov/eco-research/ecoregions-north-america) and Hydrologic Unit Code (HUC) 6 drainages (https://water.usgs.gov/GIS/huc.html). For the latter we combined smaller watersheds with a common outlet (e.g., the various HUC6 drainages all draining into the Ohio River) into their larger drainages (Fig. 1). While slightly nonstandard, we prefer this categorization over attempting to compare small drainages with minimal collecting effort to those with hundreds of collections. We also divided the state based on glacial maximum (Gray and Letsinger 2011). Lastly, we categorized specific sampling sites by lake or size of stream (https://www.epa.gov/waterdata).

To estimate total species richness for the state, a species rarefaction curve based on all species and samples collected was produced using the program EstimateS for Windows v. 9.1 (https://www.robertkcolwell.org/pages/estimates). In addition to the basic curve, two maximum species richness estimators were calculated. The abundance-based coverage estimator (ACE) predicted total species richness based on a proportion of rare to common species, defining “rare” as any species represented by <10 specimens. The incidence-based coverage estimator (ICE) made the same prediction, but defined “rare” as any species found in <10 samples.

To assess the importance of sampling effort in collecting species, simple linear regression models were calculated for the number of species collected from each of the primary watershed, ecoregion, glacial maximum, and waterbody type designations (dependent variable) based on the accumulated number of unique collections and samples combined (independent variable). Separate models were calculated for the pre-1983 and post-2005 time periods. The number of species associated with each geographic and habitat designation was treated as an independent observation even though each sample or collection was associated with designations of all four types.

## ﻿Results

A total of 219 caddisfly species among 18 families and 62 genera were determined to occur in the state of Indiana, including 17 species reported for the first time herein (Table 1). An additional seven species were removed from the state checklist due to misidentified specimens, taxonomic changes, or dubious identifications lacking voucher specimens (Table 2). The determined species are based on 80,298 total specimens representing 5223 species occurrence records from 711 unique collecting events before 1983 and 405 events after 2005 (Suppl. material 1). Because a detailed taxonomic history, including all synonymies, and regional distributions of all 219 species have already been treated in Houghton et al. (2022) and Rasmussen and Morse (2023), we do not reproduce those data herein.

**Table 1.** The 219 caddisfly species known to occur in Indiana based on all historical and contemporary collecting and sampling. All taxa are arranged alphabetically by order and family. Species reported from the state for the first time are in boldface font. Species records displayed based on those found before 1983 and after 2005. Rarity designation based on number of records after 2005: >20 = abundant, 6–20 = common, 1–5 = rare, 0 = data deficient to determine if the species still exists in the state. Most recent known collection year of data-deficient species are in the last column.

	Records before 1983	Records after 2005	Rarity	Most recent
BRACHYCENTRIDAE (5)				
*Brachycentruslateralis* (Say, 1823)	1	0	Deficient	1903
*Brachycentrusnumerosus* (Say, 1823)	9	4	Rare	–
*Micrasemarusticum* (Hagen, 1868)	3	4	Rare	–
*Micrasemascotti* Ross, 1947	2	0	Deficient	1977
***Micrasemawataga* Ross, 1938**	**0**	**2**	**Rare**	–
DIPSEUDOPSIDAE (2)				
*Phylocentropuslucidus* (Hagen, 1961)	1	0	Deficient	1980
*Phylocentropusplacidus* (Banks, 1905)	3	6	Common	–
GLOSSOSOMATIDAE (11)				
*Agapetusgelbae* Ross, 1947	2	0	Deficient	1946
*Agapetusillini* Ross, 1938	1	2	Rare	–
***Agapetusspinosus* Etnier & Way, 1973**	**0**	**1**	**Rare**	–
*Glossosomaintermedium* (Klapálek, 1892)	3	2	Rare	–
*Glossosomanigrior* Banks, 1911	1	6	Common	–
*Protoptilaerotica* Ross, 1938	1	13	Common	–
***Protoptilageorgiana* Denning, 1948**	**0**	**1**	**Rare**	–
***Protoptilalega* Ross, 1941**	**0**	**1**	**Rare**	–
*Protoptilamaculata* (Hagen, 1861)	7	36	Abundant	–
*Protoptilapalina* Ross, 1941	1	0	Deficient	1948
*Protoptilatenebrosa* (Walker, 1852)	1	0	Deficient	1936
GOERIDAE (1)				
*Goerastylata* Ross, 1938	1	1	Rare	–
HELICOPSYCHIDAE (1)				
*Helicopsycheborealis* (Hagen, 1861)	30	59	Abundant	–
HYDROPSYCHIDAE (38)				
*Cheumatopsycheanalis* (Banks, 1908)	44	111	Abundant	–
*Cheumatopsycheaphanta* Ross, 1938	3	4	Rare	–
*Cheumatopsycheburksi* Ross, 1941	2	17	Common	–
*Cheumatopsychecampyla* Ross, 1938	37	103	Abundant	–
*Cheumatopsychelasia* Ross, 1938	1	1	Rare	–
*Cheumatopsycheminuscula* (Banks, 1907)	1	0	Deficient	1957
*Cheumatopsycheoxa* Ross, 1938	24	58	Abundant	–
*Cheumatopsychepasella* Ross, 1941	9	49	Abundant	–
*Cheumatopsychesordida* (Hagen, 1861)	4	15	Common	–
*Cheumatopsychespeciosa* (Banks, 1904)	7	2	Rare	–
*Diplectronametaqui* Ross, 1970	2	3	Rare	–
*Diplectronamodesta* Banks, 1908	26	18	Common	–
*Homoplectradoringa* (Milne, 1936)	3	3	Rare	–
*Hydropsycheaerata* Ross, 1938	6	6	Common	–
*Hydropsychealternans* (Walker, 1852)	2	0	Deficient	1951
*Hydropsychearinale* Ross, 1938	1	1	Rare	–
*Hydropsychebetteni* Ross, 1938	31	88	Abundant	–
*Hydropsychebronta* Ross, 1938	17	71	Abundant	–
*Hydropsychecheilonis* Ross, 1938	14	31	Abundant	–
*Hydropsychecuanis* Ross, 1938	8	8	Common	–
*Hydropsychedepravata* Hagen, 1861	5	11	Common	–
*Hydropsychedicantha* Ross, 1938	9	10	Common	–
*Hydropsychefrisoni* Ross, 1938	4	11	Common	–
*Hydropsychehageni* Banks, 1905	1	0	Deficient	1950
*Hydropsycheincommoda* Hagen, 1861	44	68	Abundant	–
*Hydropsychemorosa* Hagen, 1861	43	7	Common	–
*Hydropsychephalerata* Hagen, 1861	13	23	Abundant	–
*Hydropsycheplacoda* Ross, 1941	0	1	Rare	–
*Hydropsychescalaris* Hagen, 1861	5	5	Rare	–
*Hydropsychesimulans* Ross, 1938	27	66	Abundant	–
*Hydropsycheslossonae* Banks, 1905	8	8	Common	–
*Hydropsychesparna* Ross, 1938	17	58	Abundant	–
*Hydropsychevalanis* Ross, 1938	8	1	Rare	–
*Macrostemumcarolina* (Banks, 1909)	10	11	Common	–
*Macrostemumtransversum* (Walker, 1852)	2	1	Rare	–
*Macrostemumzebratum* (Hagen, 1861)	14	11	Common	–
*Potamyiaflava* (Hagen, 1861)	46	92	Abundant	–
HYDROPTILIDAE (42)				
*Agrayleamultipunctata* Curtis, 1834	5	12	Common	–
*Dibusaangata* Ross, 1939	1	0	Deficient	1950
*Hydroptilaajax* Ross, 1938	2	19	Common	–
*Hydroptilaalbicornis* Hagen, 1861	1	2	Rare	–
*Hydroptilaamoena* Ross, 1938	1	0	Deficient	1976
*Hydroptilaangusta* Ross, 1938	8	66	Abundant	–
*Hydroptilaarmata* Ross, 1938	7	77	Abundant	–
*Hydroptilaconsimilis* Morton, 1905	6	56	Abundant	–
*Hydroptiladelineata* Morton, 1905	2	0	Deficient	1937
*Hydroptilagrandiosa* Ross, 1938	5	53	Abundant	–
*Hydroptilagunda* Milne, 1939	0	10	Common	–
*Hydroptilahamata* Morton, 1905	1	26	Abundant	–
*Hydroptilajackmanni* Blickle, 1963	1	0	Deficient	1976
*Hydroptilaperdita* Morton, 1905	10	72	Abundant	–
*Hydroptilascolops* Ross, 1938	0	2	Rare	–
*Hydroptilaspatulata* Morton, 1905	3	16	Common	–
*Hydroptilavala* Ross, 1938	1	0	Deficient	1976
*Hydroptilawaubesiana* Betten, 1934	16	128	Abundant	–
***Ithytrichiaclavata* Morton, 1905**	**0**	**4**	**Rare**	–
***Leucotrichiapictipes* (Banks, 1911)**	**0**	**1**	**Rare**	–
*Mayatrichiaayama* Mosely, 1937	1	1	Rare	–
*Neotrichiaminutisimella* (Chambers, 1873)	1	1	Rare	–
*Neotrichiaokopa* Ross, 1939	0	1	Rare	–
***Neotrichiavibrans* Ross, 1938**	**0**	**3**	**Rare**	–
*Ochrotrichiaeliaga* (Ross, 1941)	3	0	Deficient	1975
*Ochrotrichiariesi* Ross, 1944	1	0	Deficient	1945
*Ochrotrichiatarsalis* (Hagen, 1861)	6	26	Abundant	–
*Ochrotrichiawojcickyi* Blickle, 1963	1	0	Deficient	1980
*Ochrotrichiaxena* (Ross, 1938)	3	0	Deficient	1976
*Orthotrichiaaegerfasciella* (Chambers, 1873)	5	63	Abundant	–
***Orthotrichiabaldufi* Kingsolver & Ross, 1961**	**0**	**2**	**Rare**	–
*Orthotrichiacristata* Morton, 1905	5	43	Abundant	–
*Oxyethiracoercens* Morton, 1905	2	2	Rare	–
*Oxyethiradualis* Morton, 1905	0	1	Rare	–
*Oxyethiraforcipata* Mosely, 1934	1	19	Common	–
*Oxyethiragrisea* Betten, 1834	2	0	Deficient	1937
***Oxyethiranovasota* Ross, 1944**	**0**	**1**	**Rare**	–
***Oxyethiraobtatus* Denning, 1947**	**0**	**4**	**Rare**	–
*Oxyethirapallida* (Banks, 1904)	7	102	Abundant	–
*Oxyethiraserrata* Ross, 1938	0	3	Rare	–
***Oxyethirazeronia* Ross, 1941**	**0**	**8**	**Common**	–
*Stactobielladelira* (Ross, 1938)	1	1	Rare	–
LEPIDOSTOMATIDAE (3)				
***Lepidostomaliba* Ross, 1941**	**3**	**1**	**Rare**	–
*Lepidostomasommermanae* Ross, 1946	2	0	Deficient	1980
*Lepidostomatogatum* (Hagen, 1861)	0	11	Common	–
LEPTOCERIDAE (43)				
*Ceracleaalagma* (Ross, 1938)	4	12	Common	–
*Ceracleaancylus* (Vorhies, 1909)	6	5	Rare	–
*Ceracleaannulicornis* (Stephens, 1836)	1	1	Rare	–
*Ceracleacancellata* (Betten, 1934)	14	19	Common	–
*Ceracleadiluta* (Hagen, 1861)	6	0	Deficient	1975
***Ceracleaenodis* Whitlock & Morse, 1994**	**0**	**1**	**Rare**	–
*Ceracleaflava* (Banks, 1904)	3	5	Rare	–
*Ceracleamaculata* (Banks, 1899)	24	96	Abundant	–
*Ceracleamentiea* (Walker, 1852)	1	3	Rare	–
***Ceracleanepha* (Ross, 1944)**	**0**	**2**	**Rare**	–
*Ceracleaophioderus* (Ross, 1938)	1	0	Deficient	1947
*Ceracleapunctata* (Banks, 1894)	0	4	Rare	–
*Ceraclearesurgens* (Walker, 1852)	4	0	Deficient	1975
*Ceracleaspongillovorax* (Resh, 1974)	2	0	Deficient	1974
*Ceracleatarsipunctata* (Vorhies,1909)	19	90	Abundant	–
*Ceracleatransversa* (Hagen, 1861)	19	42	Abundant	–
*Leptocerusamericanus* (Banks, 1899)	20	82	Abundant	–
*Mystacidesinterjectus* (Banks, 1914)	4	1	Rare	–
*Mystacidessepulchralis* (Walker, 1852)	13	23	Abundant	–
*Nectopsychealbida* (Walker, 1852)	4	9	Common	–
*Nectopsychecandida* (Hagen) 1861	27	45	Abundant	–
*Nectopsychediarina* (Ross, 1944)	14	27	Abundant	–
*Nectopsycheexquisita* (Walker, 1852)	8	14	Common	–
*Nectopsychepavida* (Hagen, 1861)	6	41	Abundant	–
*Oecetisavara* (Banks, 1895)	7	27	Abundant	–
*Oecetiscinerascens* (Hagen, 1861)	27	85	Abundant	–
*Oecetisditissa* Ross, 1966	8	11	Common	–
*Oecetisinconspicua* (Walker, 1852)	46	159	Abundant	–
*Oecetisimmobilis* (Hagen, 1861)	9	1	Rare	–
*Oecetisnocturna* Ross, 1966	14	24	Abundant	–
*Oecetisochracea* Curtis, 1825	2	2	Rare	–
*Oecetisosteni* Milne, 1934	12	3	Rare	–
*Oecetispersimilis* (Banks, 1907)	7	47	Abundant	–
*Setodesoligius* (Ross, 1938)	3	2	Rare	–
*Triaenodesaba* Milne, 1935	1	15	Common	–
*Triaenodesflavescens* Banks, 1900	3	0	Deficient	1980
*Triaenodesignitus* (Walker, 1852)	3	26	Abundant	–
*Triaenodesinjustus* (Hagen, 1861)	12	50	Abundant	–
*Triaenodesmarginatus* Sibley, 1926	3	34	Abundant	–
*Triaenodesmelacus* Ross, 1947	1	16	Common	–
*Triaenodesnox* Ross, 1941	3	2	Rare	–
***Triaenodesperna* Ross, 1938**	**0**	**4**	**Rare**	–
*Triaenodestardus* Milne, 1934	17	57	Abundant	–
LIMNEPHILIDAE (20)				
*Anaboliabimaculata* (Walker, 1852)	4	2	Rare	–
*Anaboliaconsocia* (Walker, 1852)	7	3	Rare	–
*Frenesiamissa* (Milne, 1935)	5	1	Rare	–
*Hydatophylaxargus* (Harris, 1869)	5	0	Deficient	1980
*Ironoquiakaskaskia* (Ross, 1944)	1	0	Deficient	unknown
*Ironoquialyrata* (Ross, 1938)	1	0	Deficient	1978
*Ironoquiapunctatissima* (Walker, 1852)	3	10	Common	–
*Limnephilusindivisus* Walker, 1852	8	4	Rare	–
*Limnephilusornatus* Banks, 1897	2	0	Deficient	1946
*Limnephilusrhombicus* (Linneaus, 1758)	1	0	Deficient	1960
*Limnephilussubmonilifer* Walker, 1852	16	4	Rare	–
*Platycentropusradiatus* (Say, 1824)	9	11	Common	–
*Pseudostenophylaxuniformis* (Betten, 1934)	3	2	Rare	–
*Pycnopsycheguttifera* (Walker, 1852)	6	14	Common	–
*Pycnopsycheindiana* (Ross, 1938)	7	30	Abundant	–
*Pycnopsychelepida* (Hagen, 1861)	6	5	Rare	–
*Pycnopsycheluculenta* (Betten, 1934)	4	0	Deficient	1981
*Pycnopsycherossi* Betten, 1950	2	0	Deficient	1980
*Pycnopsychescabripennis* (Rambur, 1842)	9	3	Rare	–
*Pycnopsychesubfasciata* (Say, 1828)	15	17	Common	–
MOLANNIDAE (4)				
*Molannablenda* Sibley, 1926	2	0	Deficient	1981
*Molannatryphena* Betten, 1934	0	7	Common	–
*Molannaulmerina* Navas, 1934	3	0	Deficient	1960
*Molannauniophila* Vorhies, 1909	10	6	Common	–
ODONTOCERIDAE (1)				
*Mariliaflexuosa* Ulmer, 1905	2	2	Rare	–
PHILOPOTAMIDAE (7)				
*Chimarraaterrima* Hagen, 1861	10	12	Common	–
*Chimarraferia* Ross, 1941	3	9	Common	–
*Chimarramoselyi* Denning, 1948	1	0	Deficient	unknown
*Chimarraobscura* (Walker, 1852)	8	98	Abundant	–
*Dolophilodesdistinctus* (Walker, 1852)	6	6	Common	–
*Wormaldiamoesta* (Banks, 1914)	4	7	Common	–
*Wormaldiashawnee* (Ross, 1938)	1	2	Rare	–
PHRYGANEIDAE (11)				
*Agrypniastraminea* Hagen, 1873	2	0	Deficient	1948
*Agrypniavestita* (Walker, 1852)	6	5	Rare	–
*Banksiolacrotchi* Banks, 1943	1	6	Common	–
*Fabriainornata* (Banks, 1907)	1	0	Deficient	1966
*Oligostomisocelligera* (Walker, 1852)	1	0	Deficient	1978
*Phryganeacinerea* Walker, 1852	1	4	Rare	–
*Phryganeasayi* Milne, 1931	3	4	Rare	–
*Ptilostomisangustipennis* (Hagen, 1873)	1	0	Deficient	1950
*Ptilostomisocellifera* (Walker, 1852)	7	28	Abundant	–
*Ptilostomispostica* (Walker, 1852)	4	4	Rare	–
*Ptilostomissemifasciata* (Say, 1828)	2	9	Common	–
POLYCENTROPODIDAE (20)				
*Cernotinacalcea* Ross, 1938	0	15	Common	–
*Cernotinaspicata* Ross, 1938	4	24	Abundant	–
*Cyrnellusfraternus* (Banks, 1913)	17	67	Abundant	–
*Holocentropusflavus* Banks, 1909	1	0	Deficient	1981
*Holocentropusglacialis* Ross, 1938	5	4	Rare	–
*Holocentropusinterruptus* Banks, 1914	4	1	Rare	–
*Neureclipsiscrepuscularis* (Walker, 1852)	18	50	Abundant	–
*Neureclipsispiersoni* Frazer & Harris, 1991	1	2	Rare	–
*Nyctiophylaxaffinis* (Banks, 1897)	9	12	Common	–
*Nyctiophylaxmoestus* Banks, 1911	5	57	Abundant	–
*Plectrocnemiacinerea* (Hagen, 1861)	20	48	Abundant	–
*Plectrocnemiaclinei* (Milne, 1936)	0	1	Rare	–
*Plectrocnemiacrassicornis* (Walker, 1852)	2	3	Rare	–
*Plectrocnemianascotius* (Ross, 1941)	0	4	Rare	–
*Plectrocnemiaremotus* Banks, 1911	4	2	Rare	–
*Polycentropuscentralis* Banks, 1914	7	24	Abundant	–
*Polycentropuschelatus* Ross & Yamamoto, 1965	1	0	Deficient	1976
*Polycentropusconfusus* Hagen, 1861	0	12	Common	–
*Polycentropuselarus* Ross, 1944	1	0	Deficient	1963
***Polycentropuspentus* Ross, 1941**	**0**	**1**	**Rare**	–
PSYCHOMYIIDAE (2)				
*Lypediversa* (Banks, 1914)	3	42	Abundant	–
*Psychomyiaflavida* Hagen, 1861	3	37	Abundant	–
RHYACOPHILIDAE (6)				
*Rhyacophilafenestra* Ross, 1938	6	15	Common	–
*Rhyacophilaglaberrima* Ulmer, 1907	1	0	Deficient	1948
*Rhyacophilaledra* Ross, 1939	5	4	Rare	–
*Rhyacophilalobifera* Betten, 1934	7	20	Common	–
*Rhyacophilaparantra* Ross, 1948	6	1	Rare	–
*Rhyacophilavibox* Milne, 1936	1	2	Rare	–
THREMMATIDAE (3)				
*Neophylaxayanus* Ross, 1938	2	4	Rare	–
*Neophylaxconcinnus* MacLachlan, 1871	13	22	Abundant	–
*Neophylaxfuscus* Banks, 1903	3	0	Deficient	1958
Total records	1399	3824		–
Total genera	60	59		–
Total species	191	175		–

**Table 2.** The seven species listed as occurring in Indiana (Rasmussen and Morse 2023) that should be removed from the state checklist due to misidentified specimens, taxonomic changes, or dubious identification without voucher specimens.

Taxon	Reference	Reason
*Cheumatopsycheharwoodi* Denning, 1948	Waltz and McCafferty 1983	Misidentified. Specimens are actually *C.analis*
*Hydropsychealvata* Denning, 1949	Waltz and McCafferty 1983	Junior synonym of *H.incommoda* (Korecki 2006
*Hydropsychebidens* Ross, 1938	Waltz and McCafferty 1983	Junior synonym of *H.incommoda* (Korecki 2006)
*Hydropsycheorris* Ross, 1938	Waltz and McCafferty 1983	Junior synonym of *H.incommoda* (Korecki 2006)
*Hydropsycherossi* Flint et al., 1979	Waltz and McCafferty 1983	Junior synonym of *H.simulans* (Korecki 2006)
*Hydropsychevenularis* Banks, 1914	Bright (1985)	Larval record without voucher specimens
*Pycnopsycheantica* (Walker, 1852)	Wojtowicz (1982)	Junior synonym of *P.scabripennis* (Green 2023)

**Figure 3.**
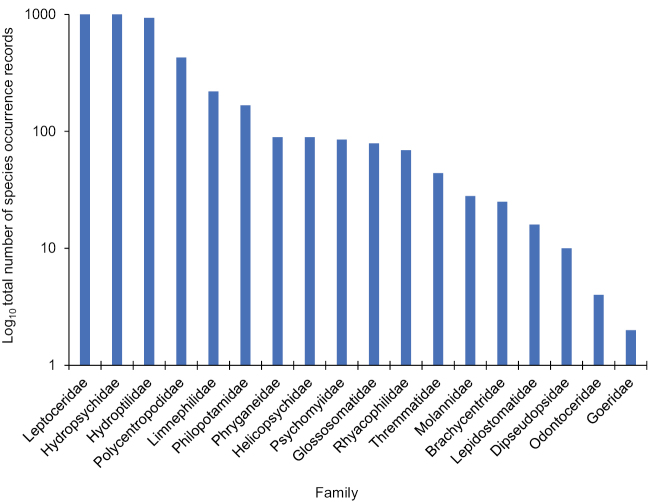
Log_10_ number of species occurrence records for each of the 18 caddisfly families known from Indiana based on all historical and contemporary collecting and sampling.

**Figure 4.**
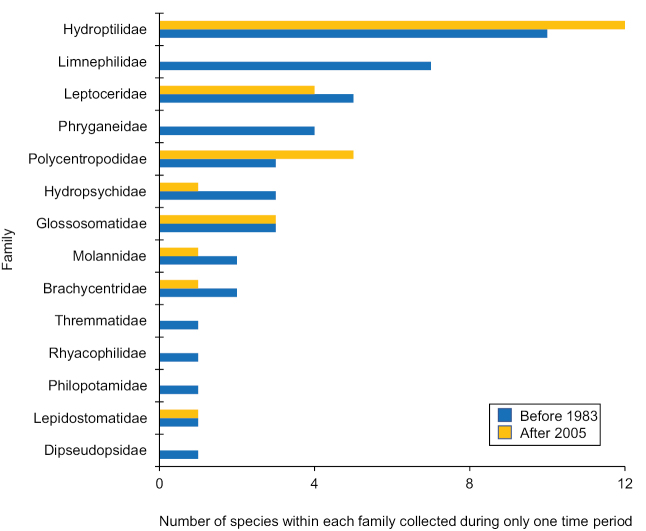
The 72 species collected either before 1983 or after 2005, but not during both periods, organized by family.

Of the known species, 100 (46%) were considered abundant or common, whereas 75 (34%) were considered rare, and 44 (20%) have not been collected in the last 40 years and, thus, were considered data deficient (Table 1). Leptoceridae (43 species), Hydroptilidae (42), and Hydropsychidae (38) were the most species rich families. They were also the families with the greatest number of total species occurrence records, collectively encompassing nearly 75% of all such records (Fig. 3). Species found only either before 1983 or after 2005 occurred in similar proportions for most families. The exceptions were the Limnephilidae and Phryganeidae, which collectively included 11 species found only before 1983 and none found only after 2005 (Fig. 4). The genera *Fabria*, *Oligostomis* (both Phryganeidae), and *Hydatophylax* (Limnephilidae) were found only before 1983, whereas *Ithytrichia* and *Leucotrichia* (both Hydroptilidae) were found only after 2005.

**Figure 5.**
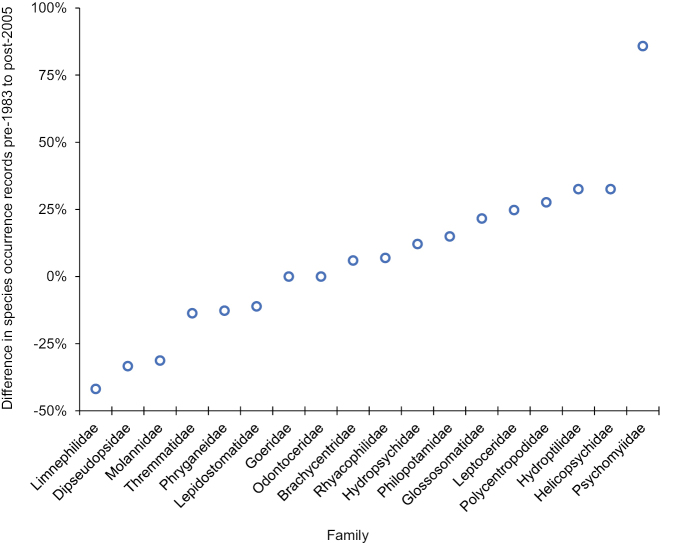
Mean difference between the two time periods of the study in the number of total species occurrence records among the 18 caddisfly families known from Indiana. Difference per species was calculated by subtracting the number of pre-1983 records from the number of post-2005 records and then dividing the result by the total number of records. These values were then averaged to determine the mean difference per family. A positive value signified a greater number of post-2005 records, whereas a negative value signified a greater number of pre-1983 records. Species occurrence data taken from Table 1.

**Figure 6.**
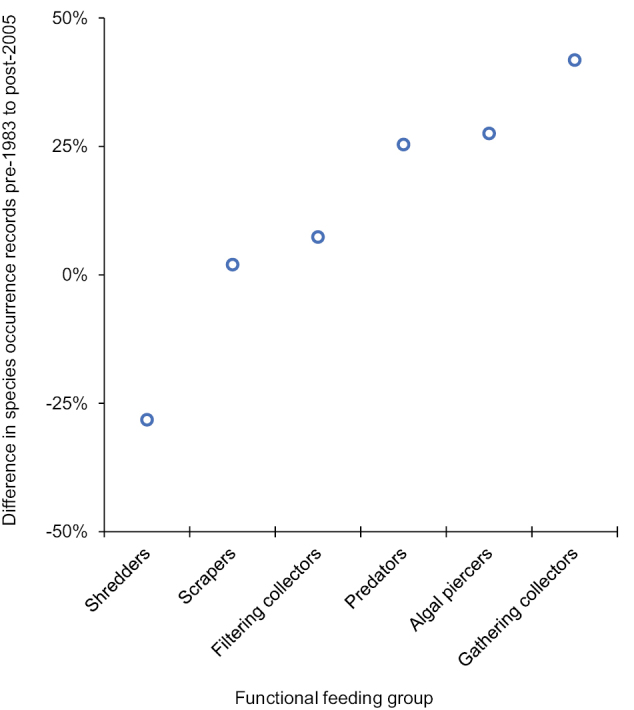
Mean difference between the two time periods of the study in the number of total species occurrence records among the five primary functional feeding groups (FFGs) known from Indiana. Difference per species was calculated by subtracting the number of pre-1983 records from the number of post-2005 records and then dividing the result by the total number of records. These values were then averaged to determine the mean difference per FFG. A positive value signified a greater number of post-2005 records, whereas a negative value signified a greater number of pre-1983 records. Species occurrence data taken from Table 1. FFG data taken from Merritt et al. (2019).

On average, species for 12 of the 18 families had an equal or greater number of occurrence records after 2005 than they did before 1983. The exceptions were the Lepidostomatidae (−11%), Phryganeidae (−12%), Thremmatidae (−13%), Molannidae (−31%), Dipseudopsidae (−33%), and Limnephilidae (−42%) (Fig. 5). Similarly, all FFGs had an equal or greater number of occurrence records after 2005 than they did before 1983, except for shredders which decreased by nearly 30% (Fig. 6).

**Figure 7.**
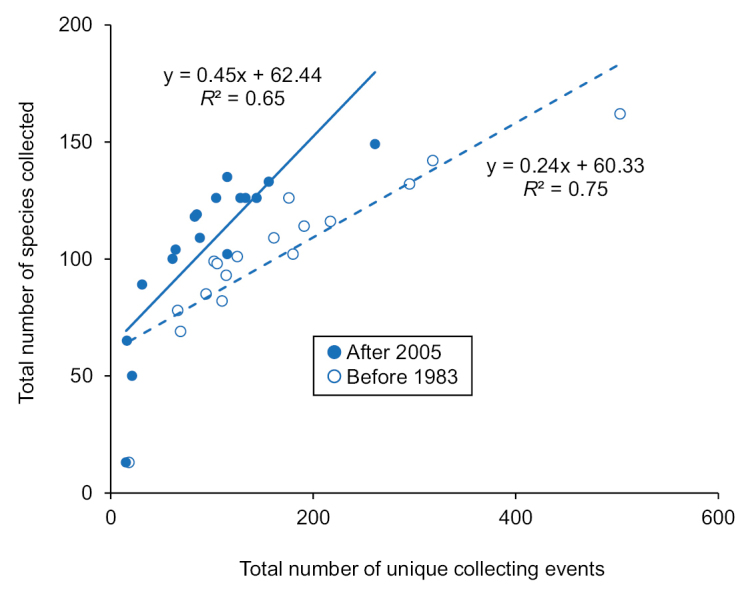
Simple linear regression models of caddisfly species richness (dependent variable) based on the total number of combined collections and samples taken (independent variable) for the two time periods of the study based on all geographic and ecological subunits of Indiana (Suppl. material 2).

**Figure 8.**
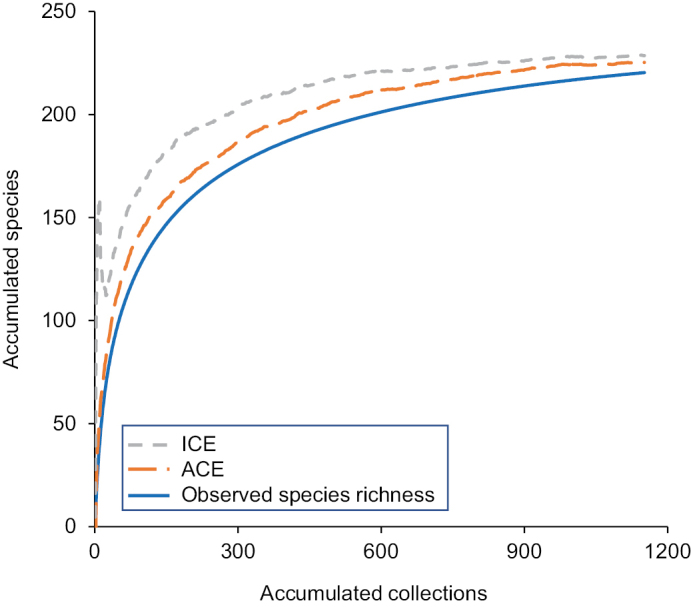
Species rarefaction curves for all historical and recent collections and samples, showing the accumulated number of species and two estimators: the abundance-based coverage estimator (ACE) and the incidence-based coverage estimator (ICE) of actual species richness. For each series, 50 randomized combinations of sample order were calculated and a mean value determined and displayed.

Individual associations between species and the various geographic and habitat designations are in Suppl. material 2 and summarized in Suppl. material 1. Overall species richness differences between the different designations were unremarkable, with the number of unique collecting events being a strong predictor of species richness for both pre-1983 and post-2005 time periods (Fig. 7). Fewer species were caught after 2005 (175) than before 1983 (191) despite having nearly 3× the species occurrence records in the post-2005 time period (Table 1). Total species richness for Indiana was predicted to be 225 and 228 species by ACE and ICE respectively (Fig. 8).

## ﻿Discussion

Overall species richness within the state was not particularly remarkable or regionally distinctive, which probably reflected a general lack of habitat diversity within Indiana relative to nearby states like Michigan or Wisconsin (Omernik and Griffith 2014). Indiana has no known endemic caddisflies (Rasmussen and Morse 2023). Total species richness of Indiana lagged behind that of the adjacent states of Michigan (319 species), Kentucky (296), Wisconsin (284), and Ohio (276), but was slightly ahead of Illinois (218) (Houghton et al. 2022). Perhaps the most noteworthy difference was the higher richness in the northern half of the state despite having higher agricultural disturbance than the southern half. The Lake Michigan watershed was particularly rich despite having one of the smallest areas. This difference may be due to the high sampling effort of the region. It may also be that the northern portion of Indiana has naturally high species richness due to naturally high groundwater input or its position as an ecotone between prairie and forest (Omernik and Griffith 2014; DeWalt et al. 2016b). In the absence of disturbance, Houghton and DeWalt (2023) predicted the Wisconsin glaciated area in the northern region of the state to have ~1.5× the caddisfly richness per stream than the Illinoian or unglaciated areas. The age of the habitats might also be important, as the more heterogeneous substrates left behind by the recent Wisconsin glaciation probably increased the microhabitat diversity of streams relative to the older eroded landscapes of the Illinoian and unglaciated regions (Benn and Evans 2010).

Differences in caddisfly species occurrence records between the pre-1983 and post-2005 sampling periods indicated the effects of continued habitat degradation in the state. The goal of the current study was to sample the caddisflies with a greater effort than had been done during the pre-1983 sampling period. It is difficult to state definitively that this goal has been accomplished due to the unclear effort of pre-1983 collections; however, the almost 3× greater number of species occurrence records overall and for most families and FFGs in the post-2005 sampling period suggested that it has. Most exceptions were species that were physically large, such as those of Limnephilidae, Molannidae, and Phryganeidae, and in the shredder FFG, such as those of Lepidostomatidae, Limnephilidae, and Phryganeidae. The other two decreasing families, Dipseudopsidae and Thremmatidae have only a few species and, thus, may be more prone to stochastic variation. Houghton and Holzenthal (2010) noted a similar decrease in species occurrence records for large shredders in the Limnephilidae and Phryganeidae in Minnesota. In a study of the Upper Midwest region of the USA, Houghton and DeWalt (2021) observed that >50% of richness loss in shredder species was explained by watershed disturbance, which was more than that of any other FFG. Since shredders are directly dependent on the input of their coarse allochthonous food source, it is expected that they would most directly correlate with intact habitat, especially that of the riparian zone (Houghton et al. 2011; Dohet et al. 2014; Entrekin et al. 2020; Houghton 2021; Williams and Houghton 2024). Moreover, larger caddisfly species in the Limnephilidae and Phryganeidae tend to be uni- or semivoltine (Merritt et al. 2019) and their longer larval period would expose them to habitat disturbances for more time than a multivoltine species would experience. Such a phenomenon has been previously noted for stoneflies in Illinois (DeWalt et al. 2005).

Collection data for new state species records are in Suppl. material 3. The majority of these records are not surprising, as they have previously been found in at least one state adjacent to Indiana. The two notable exceptions were *Agapetusspinosus* Etnier & Way, 1973 and *Protoptilageorgiana* Denning, 1948 (both Glossosomatidae). Both of these species were previously thought to be endemic to the southeastern USA, with *A.spinosus* known only from Alabama, South Carolina, and Tennessee, and *P.georgiana* from Alabama, Georgia, Maryland, North Carolina, and Virginia (Rasmussen and Morse 2023). Interestingly, both species were collected from the same site: Marble Creek, downstream of the Big Oaks Wildlife Refuge (BONWR) in Jefferson County (38.8983, −85.4646). The BONWR is one of the least disturbed habitats in Indiana and also one of the least studied, with no known previous collections from it.

Due to the recent sampling effort, most known Indiana species are still presumed extant in the state. Nonetheless, 44 species have not been seen in >40 years and remain data deficient. Eighteen of these species have not been collected in the state since the 1950s and, thus, could have been extirpated by the agricultural development that began after World War II (Omernik 1987). Most notably, *Brachycentruslateralis* (Say, 1823) has not been seen in Indiana for 121 years.

Future research should include additional sampling. While the species rarefaction curve only predicts a few more species to be found in Indiana, the strong relationship between sampling effort and species caught within the various geographic and habitat designations suggests that a “Wallacean Shortfall” – a lack of detailed data on species distributions (Lomolino 2004) – still remains within the state, and that additional sampling is needed. This shortfall may be pronounced in some autumn-emergent species of Lepidostomatidae and Limnephilidae, due to the difficulty of collecting during the autumn flight period. Since species records for both of these families have decreased since the pre-1983 time period, more autumn sampling is necessary to clarify the reason for this decrease. Conservation efforts in Indiana should probably focus on the 75 rare species, all of which have been collected during the last 2–6 years and are presumed to be extant. Specifically, more information on the life history and specific habitat needs of rare species is necessary to formulate more specific plans for their conservation. Lastly, known or suspected habitats of the 44 data-deficient species should be sampled to ascertain whether these species remain extant in Indiana.

## ﻿Additional information

### Author ORCIDs

David Houghton https://orcid.org/0000-0001-6946-4864

R. Edward DeWalt https://orcid.org/0000-0001-9985-9250

### Data availability

All of the data that support the findings of this study are available in the main text or Supplementary Information.

## Supplementary materials

Supplementary material 1 Summary of our collection data by ecological regions and habitat types Data type xlsx https://binary.pensoft.net/file/1160796This dataset is made available under the Open Database License (http://opendatacommons.org/licenses/odbl/1.0/). The Open Database License (ODbL) is a license agreement intended to allow users to freely share, modify, and use this Dataset while maintaining this same freedom for others, provided that the original source and author(s) are credited.David C. Houghton, R. Edward DeWalt

Supplementary material 2 Historical (before 1983), recent (after 2005), and combined species occurrence records for the 219 known Indiana caddisfly species Data type xlsx https://binary.pensoft.net/file/1160797This dataset is made available under the Open Database License (http://opendatacommons.org/licenses/odbl/1.0/). The Open Database License (ODbL) is a license agreement intended to allow users to freely share, modify, and use this Dataset while maintaining this same freedom for others, provided that the original source and author(s) are credited.David C. Houghton, R. Edward DeWalt

Supplementary material 3 Specific collection data for the new Indiana state caddisfly species records reported herein Data type xlsx https://binary.pensoft.net/file/1160798This dataset is made available under the Open Database License (http://opendatacommons.org/licenses/odbl/1.0/). The Open Database License (ODbL) is a license agreement intended to allow users to freely share, modify, and use this Dataset while maintaining this same freedom for others, provided that the original source and author(s) are credited.David C. Houghton, R. Edward DeWalt
